# Development of a loop-mediated isothermal amplification method to rapidly detect porcine circovirus genotypes 2a and 2b

**DOI:** 10.1186/1743-422X-9-318

**Published:** 2012-12-27

**Authors:** Xiaohuo Qiu, Tian Li, Guorui Zhang, Jingjing Cao, Yulan Jin, Gang Xing, Min Liao, Jiyong Zhou

**Affiliations:** 1 Key Laboratory of Animal Virology of Ministry of Agriculture, College of Animal Sciences, Zhejiang University, 866 Yuhangtang Road, Hangzhou, 310058, PR China; 2 Zhejiang Tongdian Biotechnology Co., Ltd, Hangzhou, 310030, PR China

## Abstract

**Background:**

Porcine circovirus type 2 (PCV2), is nowadays associated with a number of diseases known as porcine circovirus-associated diseases (PCVAD), especially postweaning multisystemic wasting syndrome (PMWS). The epidemiological investigation of PCV2 infection was usually conducted by PCR, nested PCR, PCR-RFLP, TaqMan-based assay and nucleotide sequencing. However, there is still no rapid, sensitive and practical method for detecting PCV2 genotypes. As a novel nucleic acid amplification method, the loop-mediated isothermal amplification method (LAMP) has been used to detect a variety of pathogenic microorganisms.

**Results:**

Herein, a LAMP method is developed to detect the genotypes of PCV2. The diagnostic sensitivity of LAMP is 1 copy/reaction for differentiating genotypes PCV2a and PCV2b. The reaction process was completed at 65°C for 1 hour in a water bath. Cross-reactivity assay shows that this method is specific for PCV2a and PCV2b and no reactive for PCV2c and other swine-origin viruses (i.e. CSFV, PRRSV, BVDV, TGEV and PEDV, etc). Identity between LAMP and nested PCR was 92.3% on 52 field clinical samples.

**Conclusions:**

LAMP method provides a rapid, sensitive, reliable way to detect PCV2a and PCV2b, and a better means for the large scale investigation of PCV2a and PCV2b infection.

## Background

Porcine circovirus type 2 (PCV2), a small, nonenveloped, single stranded DNA virus with a rather small circular genome [1], is considered as the primary etiological agent of porcine circorvirus associated diseases (PCVAD) [2], such as postweaning multisystemic wasting syndrome (PMWS) [3,4], porcine dermatitis and nephropathy syndrome (PDNS) [5], myocarditis and abortion [6], porcine respiratory disease complex [7], etc. Since PMWS was first recognized in North America in 1991 [4], it has caused severe economic losses to the pig industry worldwide [2,8]. In 2008, the members of the EU consortium on porcine circovirus diseases proposed a standardized nomenclature for PCV2 genotype definition. By setting a phylogenetic distance threshold of 0.035 to ORF2 nucleotide sequences, PCV2 is classified into three genotypes: PCV2a, PCV2b and PCV2c [9,10]. PCV2b is the major genotype circulating in the porcine population nowadays [9,10]. PCV2c is only isolated from Danish archives [11]. Interestingly, PCV2 genotype appears a genotype shift from PCV2a to PCV2b [11,12]. Recently, PCV2d and PCV2e were also reported as two new PCV2 genotypes based on complete gemone sequence [13-15]. However new genotypes PCV2d and PCV2e were further analyzed and considered to belong to PCV2b and PCV2a [10]. These controversial PCV2 new genotypes need further investigation. At present, PCR [16], nested PCR [17], PCR-RFLP [18], TaqMan-based assay [19], nucleotide sequencing and phylogenetic analysis [12] have been used to detect and differentiate PCV2a and PCV2b. However, the application of these methods is limited somewhat by the expensive PCR equipment, the long detection period or the high technical requirements [20,21].

LAMP method with high DNA specificity was developed in 2000 as a novel nucleic acid amplification method [22]. The use of the Bst DNA polymerase with high strand displacement activity leads to a few copies of DNA can be amplified to 10^9^ in less than an hour under isothermal conditions [22]. Therefore, LAMP is simple and easy to perform once the appropriate primers are prepared. Thus far, LAMP method has been used to detect a variety of pathogenic microorganisms [23-25], including PCV2 [21,26]. However, the LAMP method used to detect PCV2 genotypes has not yet been reported. In this study, a LAMP assay was developed to provide a rapid, sensitive, reliable method for PCV2 genotypes detection.

## Results

### Optimized LAMP assay for PCV2a and PCV2b

The concentrations of the forward inner primers (FIP) and backward inner primers (BIP), deoxynucleoside triphosphates (dNTPs), betaine and the reaction temperature were optimized when the LAMP assay was developed. The optimal LAMP reaction mixture (25 μL) was composed of 0.8 μM (each) of inner primers, 0.2 μM (each) of outer primers, 0.2 mM of dNTPs, 0.5 M of betaine, 10 × ThermoPolbuffer 2.5 μL, 8U of Bst DNA polymerase, and 1 μL of template DNA. The mixture was incubated at 65°C for 1 hour, inactivated at 80°C for 10 min, and characterised a bolder ladder like bands on 2% (W/V) agarose gel under UV light after EB staining (data not shown). The similar results can also be observed by naked eyes after adding SYBR Green I dye (data not shown).

### Specificity of the LAMP assay

**Figure 1:**
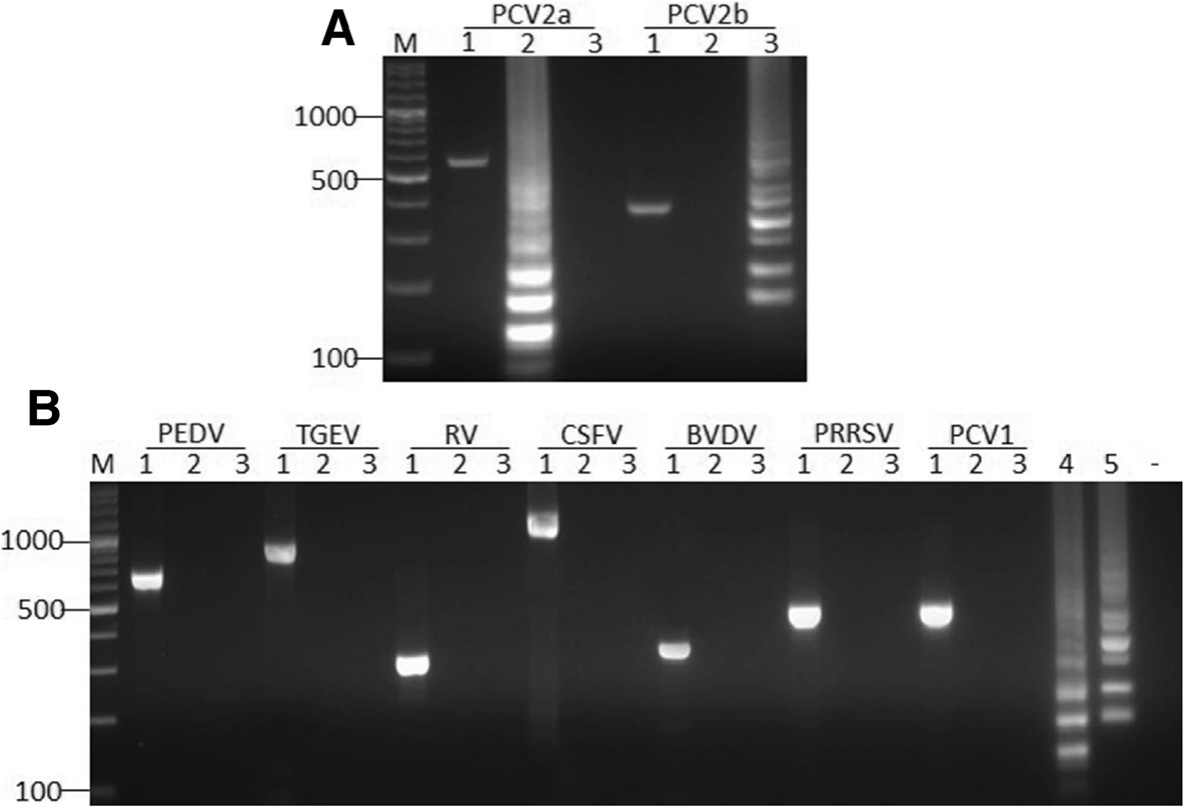
**Specificity of the LAMP method. **(**A**) Specificity of PCV2a and PCV2b. Lane M, DNA marker. Lane 1, positive control of each sample. Lane 2, postive template amplified by LAMP primers for PCV2a. Lane 3, postive template amplified by LAMP primers for PCV2b. (**B**) Cross reactivity of LAMP for PCV2 genotypes. Lane 1, positive control of each sample. Lane 2, postive template for PCV2a. Lane 3, postive template for PCV2b. Lane 4, PCV2a positive control. Lane 5, PCV2b positive control. “-”, negative control.

Data in Figure 1A showed that PCV2a and PCV2b could be detected by their special LAMP primers respectively, the same templates were amplified by nested PCR as positive control (Figure 1A, Lane 1). But the genotype PCV2c could not be detected (data not shown). Under the identical condition, none of PCV1, CSFV, PRRSV, BVDV, TGEV, PEDV and RV could give a positive reaction with the two sets of LAMP primers (Figure 1B, Lanes 2, 3), while the same templates were amplified by conventional PCR with their corresponding special primers respectively (Figure 1B, Lane 1). These data demonstrated that the LAMP assay was specific for PCV2a and PCV2b.

### Sensitivity of the LAMP assay and nested PCR assay

**Figure 2:**
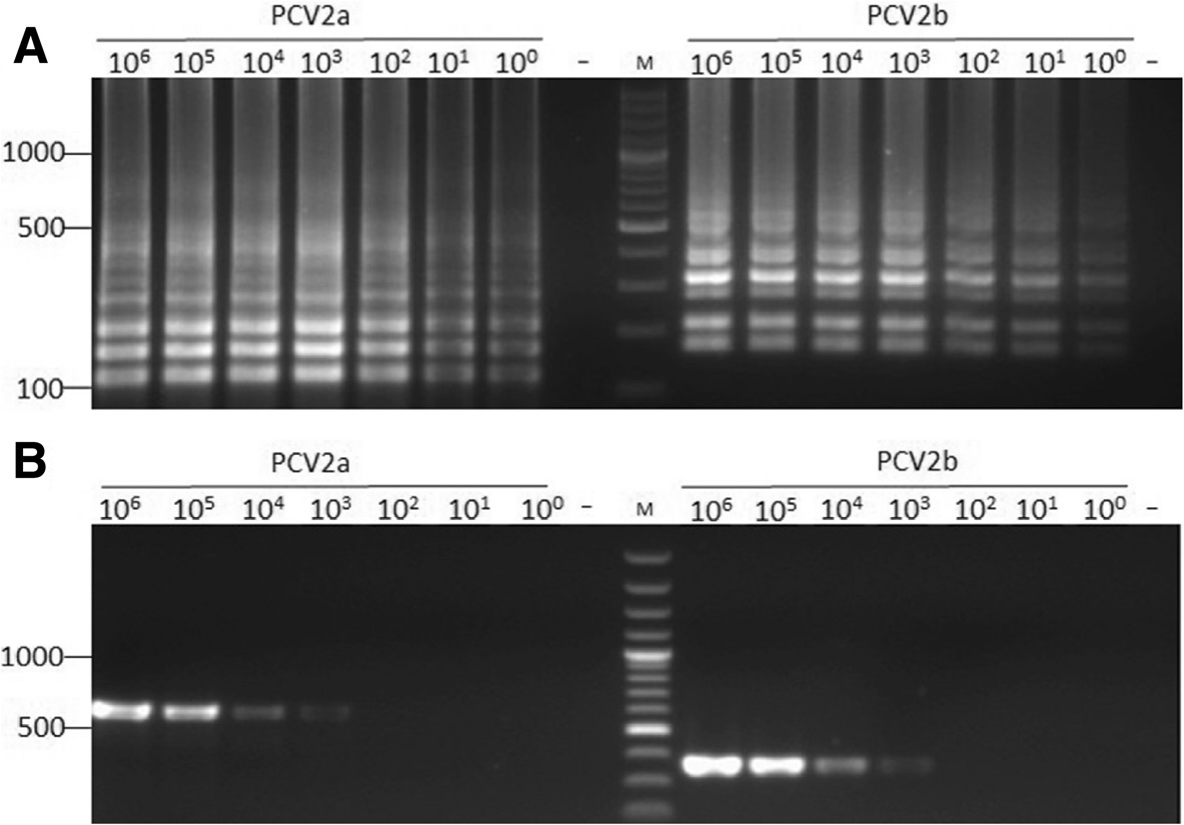
**Sensitivity of the LAMP method and nested PCR for PCV2a and PCV2b. **(**A**) Electrophoretic analysis of LAMP amplified products. (**B**) Electrophoretic analysis of nested PCR amplified products. Lane M indicates DNA Marker. 10^0^-10^6^ represents DNA copies/tube. “-” is negative control.

Sensitivity testing revealed the detection limit of the LAMP assay is 1 copy/reaction for both of PCV2a and PCV2b (Figure 2A), while the detection limit of the nested PCR was 10^3^ copies/reaction for both of PCV2a and PCV2b (Figure 2B), indicating the sensitivity of the LAMP method was elevated one thousand times in comparison with the nested PCR.

### Stability of the LAMP assay

**Figure 3:**
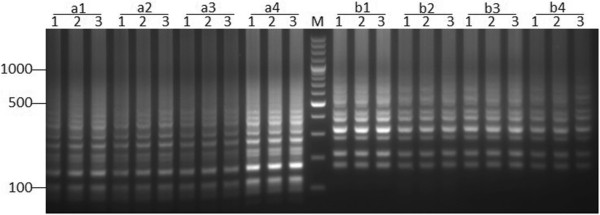
**The stability of the LAMP method for PCV2a (left side) and PCV2b (right side).** a1-a4 are four PCV2a samples. b1-b4 are four PCV2b samples. Lane M, DNA Marker. Lanes 1-3, three replications of each positive template.

Of four positive templates of PCV2a and PCV2b, three replications of each template appeared the same pattern and brightness of the amplified bands. Although the brightness of the amplified bands varied according to the different concentration of the templates, the pattern of the amplified bands is consistent. These results indicated the LAMP method was stable (Figure 3).

### PCV2a and PCV2b detection of clinical samples

Of the 52 clinical samples, 52 samples were positive for PCV2b and 4 samples were positive for genotypes PCV2a in the LAMP method, indicating that PCV2b is the major epidemic genotype and co-infection of PCV2a and PCV2b in pig farms. In the nested PCR amplification, 48 of 52 samples were positive for PCV2b and no PCV2a was detected. These data showed that the identity between LAMP and nested PCR was 92.3% (48/52) and the LAMP method had higher sensitivity than the nested PCR method.

## Discussion

Generally, the nucleotide sequencing has been used to detect PCV2a and PCV2b [12], other approaches included PCR [16], nested PCR [17], PCR-RFLP [18] and TaqMan-based assay [27]. However, these methods were usually time consuming and high technical requirement, and were difficult to popularize in veterinary clinic practice. In the present study, the LAMP method was developed for detecting PCV2 genotypes, and it was a simple, rapid and easy operation for veterinarians.

The specific genotype primers for differential PCR amplification with high sensitivity and high specificity are not easy to design, because the genetic identity between PCV2a and PCV2b is very high (>95%) [28]. However, the nucleotides sites 1480–1469 of PCV2a and PCV2b are ACCAACAAAAT and TCAAACCCCCG(T), respectively [12], therefore, we can distinguish PCV2a and PCV2b according to this tag motifs. In this study, the special LAMP primers were designed respectively based on the tag motifs of PCV2a’s AT rich area and PCV2b’s GC rich area of the ORF2 gene, and the homology of all these primer sequences among 372 PCV2 strains is over 90%, indicating that these primers could deal with most of the PCV2 strains in LAMP assay. During the development of the LAMP assay, the concentration of outer primers is fixed, and 0.4 μM, 0.6 μM, 0.8 μM is selected for optimizing the concentration of inner primers, because the outer and inner primers are used in the initial steps of the LAMP reaction and the inner primers are used for strand displacement DNA synthesis at late stage of nucleic acid amplification [22]. It has been reported that the addition of 0.5-1.6 M betaine improved the amplification of DNA by reducing the formation of secondary structure in GC-rich regions [29], and 0.5 M, 1 M, 1.5 M was selected for the concentration of betaine optimization. In addition, we found that the efficiency of nucleic acid amplification falled as the concentration of dNTPs increased, and even no products. This may be attributed to that the excessive dNTPs resulted in chelation of magnesium ions in the reaction buffer.

## Conclusions

Take together, we can find that the LAMP method has better specificity, higher sensitivity and efficiency, and simpler equipment requirements than the nested PCR. Furthermore, the detection of the amplification product is more flexible. Therefore, the LAMP method developed in this study provides a better means for the molecular epidemiological investigation of PCV2 genotypes.

## Material and methods

### Viral strains and reagents

Porcine circovirus type 1 (PCV1), porcine circovirus genotype 2a (PCV2a), porcine circovirus genotype 2b (PCV2b), classical swine fever virus (CSFV), porcine respiratory and reproductive syndrome virus (PRRSV), bovine viral diarrhea virus (BVDV) were kept in our laboratory. Transmissible gastroenteritis virus (TGEV), porcine epidemic diarrhea virus (PEDV) and rotavirus (RV) came from purchased commercial triple attenuated vaccine. Bst DNA polymerase and 10 × ThermoPolbuffer (20 mM Tris–HCl, 10 mM KCl, 10 mM (NH_4_)_2_SO_4_, 2 mM MgSO_4_, 0.1% Triton X-100) were purchased from New England Biolabs (Massachusetts, USA). Betaine was purchased from Sigma-Aldrich Co. (Missouri, USA). SYBR Green I dye was obtained from Solar Biotechnology (Beijing, China).

### Clinical specimens

Clinical specimens for inspection, including inguinal lymph nodes, mesenteric lymph nodes, livers and lungs, were collected from different pig farms in Zhejiang Province and its neighbour areas under the condition of the farmer’s consents. All tissue samples were homogenized with a mortar in minimal essential medium at the ratio of 1:10. Then the suspension was centrifuged at 6,000 rpm for 10 min to obtain the supernatant. As a positive control, PK-15 cells infected respectively with PCV1 or PCV2a or PCV2b were frozen and thawed three times, then centrifuged at 6,000 rpm for 10 min to obtain cell free supernatant. All samples were stored at -80°C until used.

### DNA and RNA extraction

The viral DNA was extracted by using the UNIQ-10 viral DNA extraction kit according to manufacturer’s instructions (Sangon Biotech Co., Ltd., Shanghai, China). RNA isolation of CSFV, PRRSV, BVDV, TGEV, PEDV and RV was carried out using Trizol Reagent (Invitrogen, USA) according to manufacturer’s specification. The extracted RNA was used as template after reverse transcription with random primer according to the instructions of the RevertAid™ First Strand cDNA Synthesis Kit (Fermentas, Canada). All templates were stored at -20°C until used.

### LAMP primers design

According to the reference sequences of PCV2a (GenBank:AF055382) and PCV2b (GenBank:AF055394) [10], two sets of primers special for PCV2a (inner primers FIPa and BIPa, outer primers F3a and B3a) and PCV2b (inner primers FIPb and BIPb, outer primers F3b and B3b) (Table 1) are designed based on the tag motif [12] in PCV2a’s AT rich area and PCV2b’s GC rich area of the ORF2 gene online (http://primerexplorer.jp/eLAMP3.0.0/index.html). Inner primers contain sequences of the sense and antisense strands of the target DNA linked by a TTTT spacer (Hightlighted in bolder pattern in Table 1). The homology of each primer among 372 PCV2 strains is calculated by MEGA (VERSION:5.0) [30]. Primers are synthesized by Sangon (Sangon Biotech Co., Ltd, Shanghai, China).

**Table 1:** Primer sets for PCV2a and PCV2b of LAMP method

**Primers for PCV2a**		**Primes for PCV2b**	
Primer name	Sequence (5′ → 3′)	Primer name	Sequence (5′ → 3′)
FIPa	AGGGCCAGAATTCAACCTTAACC**TTTT**GGGACCAACAAAATCTCT	FIPb	AGCAGGGCCAGAATTCAACCTT**TTTT**CTCAAACCCCCTCACTG
BIPa	GGGCTCCACTGCTGTTAT**TTTT**GGTCATAGGTTAGGGCTGT	BIPb	GGTGACAGGGGAGTGGGCT**TTTT**GGTCATAGGTGAGGGCTGT
F3a	TTTAAAATTGACGACTTTGTTCC	F3b	ACTTTCTTCCCCCAGGAGG
B3a	ATTGTATGGCGGGAGGAG	B3b	GGTATGGCGGGAGGAGTA

### The optimization of the LAMP method

The LAMP reaction mixture contains inner primers (FIP/BIP), outer primers (F3/B3), dNTPs, betaine, buffer, Bst DNA polymerase and the template DNA. To optimize the LAMP reaction, final concentration of FIP/BIP at 0.4 μM, 0.6 μM, 0.8 μM, dNTPs at 0.2 mM, 0.4 mM, 0.6 mM and betaine at 0.5 M, 1 M, 1.5 M, were selected to test the best reaction condition in a total of 25 μL reaction volume. In addition, the reaction temperature was selected to test the optimized LAMP reaction condition. The LAMP reaction was carried out in a water bath for 1 h, and then heated at 80°C for 10 min to terminate the reaction. The amplified products (5 μL) were electrophoresed on 2.0% (W/V) agarose gel and visualized under UV light after EB staining.

### Nested PCR

The nested PCR method for detecting PCV2a and PCV2b was developed by Lyoo in 2008 [17], and it was adjusted and used to compare with the LAMP method on sensitivity. The primer synthesis and the PCR procedure were followed as described in the paper [17]. The polymerase, dNTPs, PCR buffer were replaced by the 2 × Taq PCR MasterMix (TIANGEN Biotech Co., Ltd, Beijing, China).

### Specificity and sensitivity

To detect the specificity of the LAMP method, PCV2a, PCV2b, PCV1, CSFV, PRRSV, BVDV, TGEV, PEDV and RV templates were amplified with LAMP primers for PCV2a and PCV2b respectively. As compared, all these templates were detected by conventional PCR with their special primers to ensure the template for individual virus is testable.

To detect the sensitivity of the LAMP method, the concentration of the extracted DNA of PCV2a and PCV2b was quantified with ND-1000 spectrophotometer (NanoDrop, Wilmington, USA), and serial ten-fold dilution method was used to dilute it from 2.5 × 10^7^ copies/μL to 2.5 × 10^1^ copies/μL.

### Stability

Four PCV2a and PCV2b postive templates were chosen to test the stability of the LAMP method, respectively. Each template was repeated three times under identical conditions. The amplified products (5 μL) were analyzed by agarose gel electrophoresis.

## Competing interests

The authors declare that they have no competing interests.

## Authors’ contributions

XQ, TL, GZ and JZ designed this experiment. XQ, JC carried out the experiments. XQ, GZ, ML and JZ wrote the manuscript. YJ and GX provided clinical samples. JZ has given final revision and approval of the version to be published. All authors read and approved the final manuscript.
